# Molecular prevalence and risk factors associated with tick-borne pathogens in cattle in western Kenya

**DOI:** 10.1186/s12917-021-03074-7

**Published:** 2021-11-27

**Authors:** Tatenda Chiuya, Jandouwe Villinger, Daniel K. Masiga, Dickens O. Ondifu, Maurice K. Murungi, Lillian Wambua, Armanda D. S. Bastos, Eric M. Fèvre, Laura C. Falzon

**Affiliations:** 1 International Centre of Insect Physiology and Ecology (icipe), P.O Box 30772-00100, Nairobi, Kenya; 2 Department of Zoology and Entomology, University of Pretoria, Private Bag 20, Pretoria, 0028 South Africa; 3 International Livestock Research Institute, Old Naivasha Road, P.O Box 30709, Nairobi, 00100 Kenya; 4 Institute of Infection, Veterinary and Ecological Sciences, University of Liverpool, Leahurst Campus, Chester High Road, Neston, CH64 7TE UK

**Keywords:** Livestock markets, Slaughterhouses, Dual infection, *Anaplasma*, *Theileria*, Western Kenya

## Abstract

**Background:**

Tick-borne pathogens (TBPs) are of global importance, especially in sub-Saharan Africa where they represent a major constraint to livestock production. Their association with human disease is also increasingly recognized, signalling their zoonotic importance. It is therefore crucial to investigate TBPs prevalence in livestock populations and the factors associated with their presence. We set out to identify TBPs present in cattle and to determine associated risk factors in western Kenya, where smallholder livestock production is important for subsistence and market-driven income.

**Results:**

Tick-borne pathogen infections in blood samples collected from cattle at livestock markets and slaughterhouses between May 2017 and January 2019 were identified by high-resolution melting analysis and sequencing of PCR products of genus-specific primers. Of the 422 cattle sampled, 30.1% (127/422) were infected with at least one TBP, while 8.8% (37/422) had dual infections. *Anaplasma* spp. (19.7%) were the most prevalent, followed by *Theileria* (12.3%), *Ehrlichia* (6.6%), and *Babesia* (0.2%) spp. Sequence analysis of the TBPs revealed them to be *Anaplasma platys*-like organisms (13.5%), *Theileria velifera* (7.4%), *Anaplasma marginale* (4.9%), *Theileria mutans* (3.1%), *Theileria parva* (1.6%), and *Babesia bigemina* (0.2%). *Ehrlichia ruminantium*, *Rickettsia* spp., and arboviruses were not detected. Exotic breeds of cattle were more likely to be infected with *A. marginale* compared to local breeds (OR: 7.99, 95% CI: 3.04–22.02, *p* <  0.001). Presence of ticks was a significant predictor for *Anaplasma* spp. (OR: 2.18, 95% CI: 1.32–3.69, *p* = 0.003) and *Ehrlichia* spp. (OR: 2.79, 95% CI: 1.22–7.23, *p* = 0.022) infection. Cattle sampled at slaughterhouses were more likely to be positive for *Anaplasma* spp. (OR: 1.64, 95% CI: 1.01–2.70, *p* = 0.048) and *A. marginale* (OR: 3.84, 95% CI: 1.43–12.21, *p* = 0.012), compared to those sampled at livestock markets.

**Conclusion:**

This study reports TBP prevalence and associated risk factors in western Kenya, factors which are key to informing surveillance and control measures.

## Background

Ticks and tick-borne pathogens (TBPs) are constraints to livestock production, causing significant economic losses to the livestock industry in sub-Saharan Africa (SSA) [1]. The major tick vectors in SSA belong to the genera *Amblyomma*, *Rhipicephalus,* and *Hyalomma*, collectively transmitting *Babesia*, *Theileria*, *Anaplasma*, *Ehrlichia, Rickettsia,* and some viral pathogens [2]. In Kenya, the commonly reported tick-borne diseases (TBDs) associated with livestock production constraints include East Coast fever (*Theileria parva*: *Rhipicephalus appendiculatus*), babesiosis (*Babesia bigemina*: *Rhipicephalus decoloratus*), anaplasmosis (*Anaplasma marginale*: *Rhipicephalus decoloratus*), and heartwater (*Ehrlichia ruminantium*: *Amblyomma variegatum*) [3–6]. Other benign *Theileria* spp., such as *Theileria taurotragi*, *Theileria mutans*, and *Theileria velifera,* are also highly prevalent [7]. Due to their endemic stability in the predominantly indigenous cattle herds in Kenya, most of these TBDs are clinically inapparent. However, exotic breeds introduced into these areas tend to manifest severe clinical diseases due to their poor innate and adaptive immune response to TBPs [8]. Pregnancy or co-infection with other pathogens may also compromise the natural resistance in indigenous cattle, leading to disease manifestation, including weight loss, a drop in milk production, and mortalities [9, 10].

While the importance of TBPs in livestock production cannot be understated, an increasing number of reports show that TBPs are zoonotic, thus posing a public health threat [11]. These zoonotic TBPs include protozoa, bacteria, and viruses. Specifically, *Babesia divergens* and *Babesia microti* cause human babesiosis, while *Ehrlichia chaffeensis* and *Anaplasma phagocytophilum* cause human ehrlichiosis and anaplasmosis, respectively [1, 12, 13]. Recent reports of *Anaplasma platys*, *Anaplasma ovis*, and *Anaplasma capra* in humans suggest that they could be of zoonotic importance [14–16]. The clinical manifestations associated with these pathogens are reported mostly from Europe and North America, and include fever, myalgia and rash [13]. In SSA, *Rickettsia africae*, which causes African tick bite fever in humans, is common, causing clinical disease in tourists and travellers [17], while only a few cases have been reported in the local population [18, 19].

Crimean-Congo haemorrhagic fever (CCHF) virus, transmitted by *Hyalomma* ticks, is one of the most widespread zoonotic tick-borne viruses globally. Clinical disease in humans is characterised by haemorrhagic fever and a mortality rate of up to 30% [20]. A single fatal human case of CCHF was reported in western Kenya in 2000 [21], and several outbreaks have been reported in neighbouring Uganda between August 2017 and January 2019, with consequent human fatalities [22]. Moreover, zoonotic *Babesia* spp., *Anaplasma* spp*.*, *Ehrlichia* spp. and *Rickettsia* spp. have been detected in cattle [23–25], and the CCHF virus was recently detected in *Rh. decoloratus* ticks from cattle at slaughterhouses in western Kenya [26]. Given that cattle are carriers of the CCHF virus [27], the role of cattle in the epidemiology of zoonotic TBPs warrants further investigation.

With an estimated 17 million cattle in different production systems, livestock, in addition to its cultural and social value, represent an important source of livelihood in Kenya [28]. In western Kenya alone, there are at least 843,608 and 219,904 indigenous and exotic cattle, respectively [29], yet only a few studies have so far been carried out to assess the prevalence and epidemiology of TBDs constraining livestock production. These studies reported a high seroprevalence of TBDs and associated risk factors in smallholder livestock production systems in the western Kenyan highlands [5] and Machakos County [3, 30, 31], while another study reported that East Coast fever was the major cause of mortality (40%) in indigenous zebu calves followed from birth to 51 weeks of age in western Kenya [32]. Another study in Lambwe Valley of western Kenya, which represents a wildlife-livestock interface, reported a high animal-level prevalence of TBPs [33], while emerging *Anaplasma* and *Ehrlichia* spp. were found to be infecting dairy cows in peri-urban Nairobi [25]. However, the presence of zoonotic TBPs as a measure of risk for human infection in this region has so far received limited attention.

Rising human population and increased demand for human habitation have resulted in increasingly fragmented landscapes and extensive interface areas, facilitating the transmission of zoonotic diseases [34, 35]. The shift to intensive and market-inclined smallholder livestock production systems being witnessed in East Africa is likely to further exacerbate the situation [36]. The livestock markets and slaughterhouses located in peri-urban areas are a conduit for the movement of livestock across internal and country borders in East Africa [37]. Given that animal trade and migration are considered important factors in the introduction and establishment of diseases in new uninfected areas [38], these facilities may be important in the epidemiology of TBDs. Therefore, we carried out this study at livestock markets (LMs) and slaughterhouses (SHs) in western Kenya to determine the prevalence of TBPs in cattle that are of animal and public health concern, using molecular analytical methods. We also determined the presence of co-infections, which may complicate diagnosis and prognosis of TBDs, and assessed which factors are associated with TBP presence in cattle.

## Results

We analyzed 422 cattle blood samples which were collected from seven LMs and SHs between May 2017 and January 2019. Of these, 219 samples were from LMs while 203 were from SHs. Most of the samples were from Kakamega County (*n* = 272) while Bungoma County (*n* = 99) and Busia County (*n* = 51) contributed the remainder. Of these, 50.9% (215/422) were from female cattle while 49.1% (207/422) were from male cattle. The selected samples were comprised of 67.8% (286/422) from local breeds, 17.5% (74/422) from cross-breed cattle, and 14.7% (62/422) from exotic breeds. Most (87.4%; 369/422) of the samples were from cattle aged 12 months and above, while 12.6% (53/422) were from those aged less than 12 months. The body condition score ranged from 1 to 2.5 in 68.5% (289/422), and from 3 to 5 in 31.5% (133/422) of the cattle. Ticks were noted and collected from 53.3% (225/422) of these cattle, while they were not observed in the remaining 46.7% (197/422) of the cattle.

### Diversity of TBPs detected by PCR-high resolution melting (HRM) analysis

**Fig. 1:**
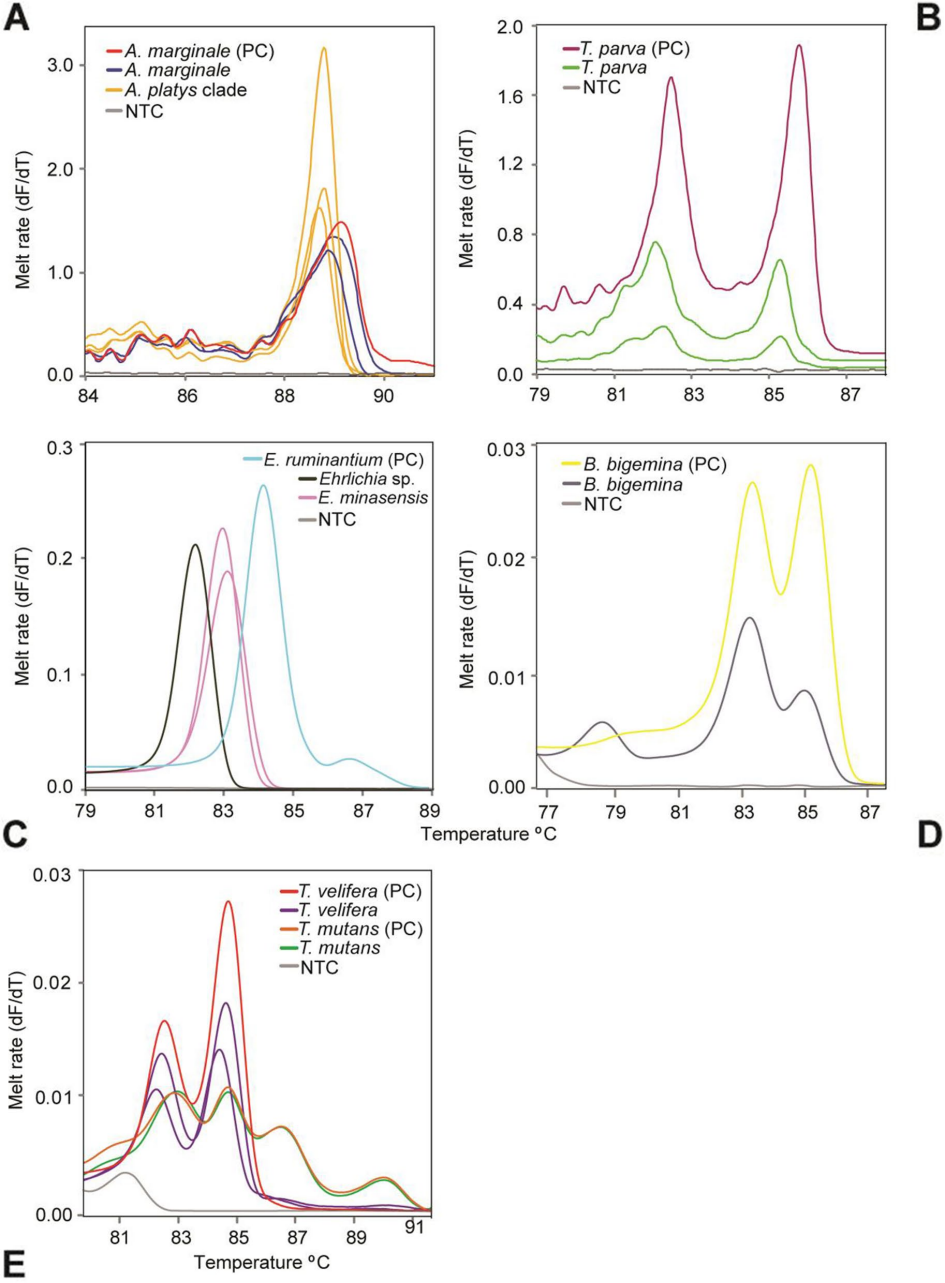
PCR amplicon melt rate profiles of representative samples. **A** *Anaplasma* spp. 16S rRNA, **B** *T. parva* 18S rRNA, **C** *Ehrlichia* spp. 16S rRNA, **D** *B. bigemina* 18S rRNA, and **E** *Theileria* spp. 18S rRNA. Positive controls are indicated by ‘PC’. No template controls are shown by ‘NTC’. Melt rates are represented as change in fluorescence with increasing temperature (dF/dT)

We detected *Anaplasma* spp., *Babesia* spp., *Ehrlichia* spp., and *Theileria* spp. by PCR-HRM among the 422 cattle blood samples analyzed. We however did not detect *E. ruminantium*, *Rickettsia* spp., or arboviruses. The identifications were based on the distinct HRM profiles and confirmatory sequencing of representative PCR amplicons as shown in Fig. 1.

**Fig. 2:**
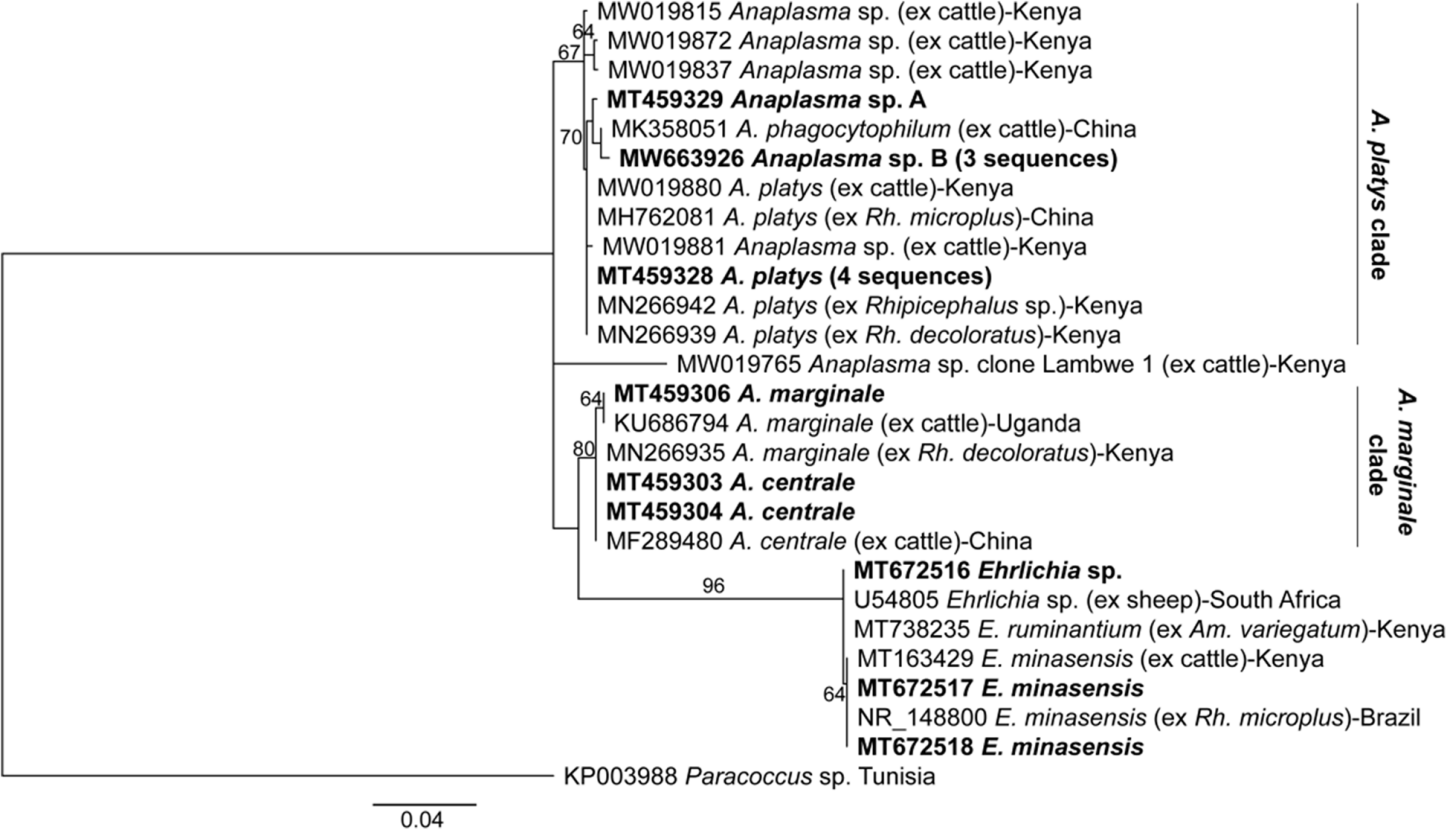
Maximum-likelihood phylogeny inferred from 26 partial 16S rRNA Anaplasmataceae sequences detected in cattle. Sequences from this study are in bold. Numbers at the nodes indicate % bootstrap support and the scale bar represents 0.03 substitutions per site

**Fig. 3:**
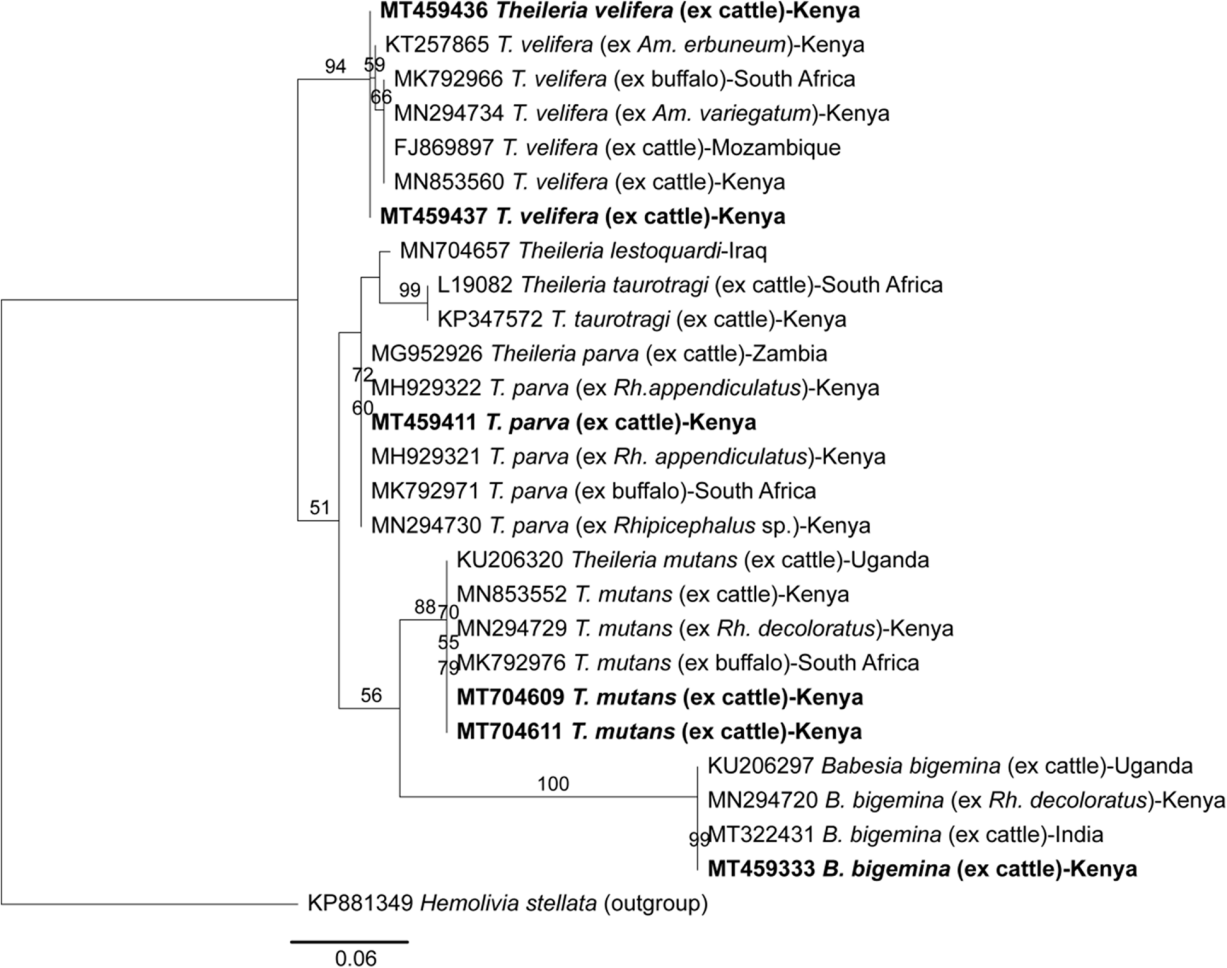
Maximum-likelihood phylogeny of 18S rRNA partial sequences of *Theileria* and *Babesia* spp. detected in cattle. Sequences from this study are in bold. Numbers at the nodes indicate % bootstrap support and the scale bar represents 0.06 substitutions per site

The phylogeny of representative sequences of pathogens identified in this study is shown in Figs. 2 and 3. The recently described *Ehrlichia minasensis* detected in this study (GenBank accessions MT672517-MT672518) clustered with *E. minasensis* detected in cattle from Kenya (GenBank accession MT163429) [25] and in *Rhipicephalus microplus* ticks from Brazil (GenBank accession NR_148800) [39]. *Anaplasma marginale* strains (GenBank accession MT459306) were closely related to those from Uganda (GenBank accession KU686794) [40], while the *Anaplasma centrale* strains (GenBank accessions MT459303-MT459304) were closely related to those from China (GenBank accession MF289480) [41]. Our *A. platys* strains (GenBank accessions MT459319-MT459321, MT459326 and MT459328) were closely related to strains previously detected in Kenya (GenBank accession MW019880) [33] and China (GenBank accession MH762081) [42]. We also detected phylogenetically diverse *A. platys*-like sequences that we classified into groups “*A*” (GenBank accession MT459329) and “*B*” (GenBank accessions MW663926-MW663928). These clustered within the *A. platys* clade and shared at least 99% identity with previously detected *A. platys* (GenBank accession MN266939) [26] and *Anaplasma phagocytophilum* (GenBank accession MK358051) [43] strains.

Our *T. parva* strains (GenBank accession MT49411) were closely related to those from South Africa (GenBank accession MK792971) [44], Zambia (GenBank accession MG952926) [45], and Kenya (GenBank MH929322) [46] and (GenBank accession MN294730) [26]. *Theileria velifera* strains (GenBank accessions MT459436-MT459437) clustered within the same clade as strains from Kenya (GenBank accession MN853560) [33] and (GenBank accession MN294734) [26], South Africa (GenBank MK792966) [44], and Mozambique (GenBank accession FJ869897) [47]. *Theileria mutans* strains (GenBank accessions MT704609; MT704611) were closely related to those from Uganda (GenBank KU206320) [48], Kenya (GenBank accessions MN853552 [33] and MN294729 [26]), and South Africa (GenBank accession MK792976) [44]. *Babesia bigemina* strains (GenBank accession MT459333) were closely related to those from Kenya (GenBank accession MN294720) [26], India (GenBank accession MT322431), and Uganda (GenBank accession KU206297) [48]. All strains of TBPs detected in this study were phylogenetically close to strains of the same pathogens previously detected in ticks collected from the same animals used in this and a prior study [26] (Figs. 2 and 3).

### Prevalence of TBPs in cattle based on PCR-HRM analysis

**Fig. 4:**
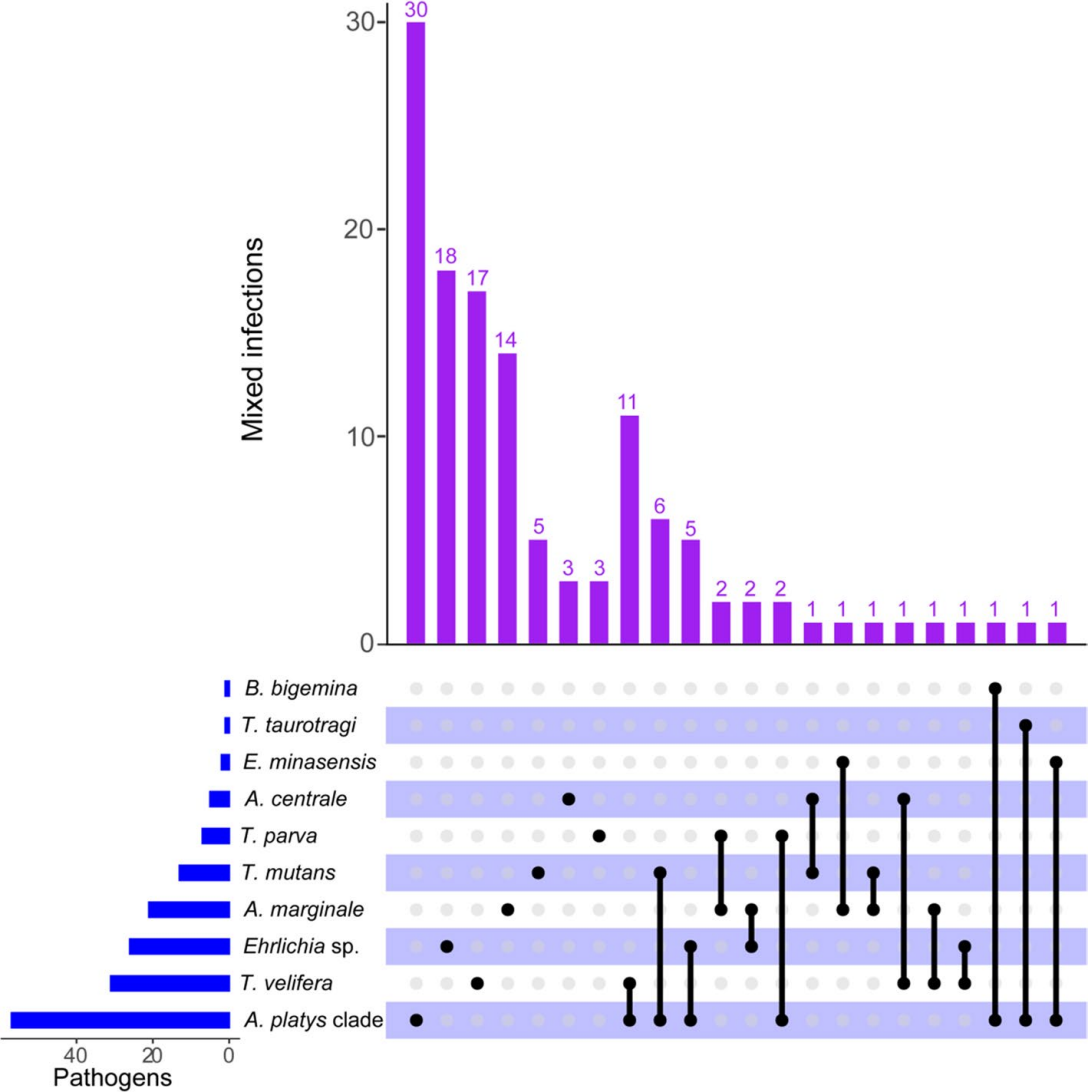
UpSetR plot showing the frequency of dual infections of tick-borne pathogens detected in cattle from western Kenya. The blue bar plot on the left shows the total number of pathogens of each species detected while the matrix shows single (black dots) and dual infections (black dots connected by black lines) whose frequency is depicted by the purple bar plot

Of the 422 cattle, 127 (30.1%) were infected with at least one TBP. Detailed information on the prevalence and infection status of the cattle is shown in Table 1 and Fig. 4. Single infections were detected in 21.3% (90/422) of the cattle and an additional 8.8% (37/422) had dual infections.

**Table 1:** Prevalence of *Anaplasma*, *Babesia*, *Ehrlichia*, and *Theileria* spp. detected in cattle from western Kenya

	Percent prevalence by County			
Tick-borne pathogen	Busia	Bungoma	Kakamega	Total
	(n = 51)	(n = 99)	(n = 272)	(***n*** = 422)
***Anaplasma*** **spp**.	**6 (11.8)**	**17 (17.2)**	**60 (22.1)**	**83 (19.7)**
*A. centrale*	0 (0)	0 (0)	5 (1.8)	5 (1.2)
*A. marginale*	0 (0)	5 (5.1)	16 (5.9)	21 (5.0)
*A. platys* clade	6 (11.8)	12 (12.1)	39 (14.3)	57 (13.5)
***Babesia*** **spp**.	**0 (0)**	**0 (0)**	**1 (0.4)**	**1 (0.2)**
*B. bigemina*	0 (0)	0 (0)	1 (0.4)	1 (0.2)
***Ehrlichia*** **spp**.	**6 (11.8)**	**5 (5.1)**	**17 (6.3)**	**28 (6.6)**
*E. minasensis*	0 (0)	1 (1.0)	1 (0.4)	2 (0.5)
*Ehrlichia* sp.	6 (11.8)	4 (4.0)	16 (5.9)	26 (6.2)
***Theileria*** **spp.**	**5 (9.8)**	**10 (10.1)**	**37 (13.6)**	**52 (12.3)**
*T. mutans*	2 (3.9)	4 (4.0)	7 (2.6)	13 (3.1)
*T. parva*	0 (0)	0 (0)	7 (2.6)	7 (1.7)
*T. taurotragi*	0 (0)	0 (0)	1(0.4)	1(0.2)
*T. velifera*	3 (5.9)	6 (6.1)	22 (8.1)	31 (7.3)

The most frequent dual infection was a combination of *T. velifera* and the *A. platys*-like organisms. The most prevalent group was *Anaplasma* spp. (19.7%), followed by *Theileria* (12.3%), *Ehrlichia* (6.6%), and *Babesia* (0.2%) spp. Kakamega County had the highest prevalence for most of the pathogens; however, this might have been biased by the selection of the samples analysed, which focused on this county due to prior detection of CCHF virus-positive ticks in a previous study [26]. At species level, detected TBP prevalences were highest for the *A. platys* clade (13.5%), *T. velifera* (7.4%), *A. marginale* (4.9%), and *T. mutans* (3.1%), with minor occurrences of other pathogens (Table 1; Fig. 4).

### Risk factor analysis

Logistic regression analysis to determine the association of predictor variables with TBP occurrence was performed at genus level for *Anaplasma* spp., *Ehrlichia* spp., and *Theileria* spp. Species level analysis was only carried out for *A. marginale* given the lack of sufficient data for the other pathogens. Furthermore, we also prioritized *A. marginale* due to its economic impact on cattle production in this region. Age as a putative risk factor was excluded from the *A. marginale* analysis due to lack of events in cattle aged less than 12 months.

Tick presence and sampling site were univariably associated with occurrence of *Anaplasma* spp., while cattle breed and sampling site were univariably associated with *Theileria* spp. and *A. marginale* occurrence. Cattle breed and tick presence were univariably associated with *Ehrlichia* spp. occurrence (Table 2).

**Table 2:** Descriptive statistics and univariable logistic regression analysis of predictor variables associated with tick-borne pathogen occurrence in cattle

Variables	Categories	Prevalence (%)	Odds ratio (95% CI)	***p***-value
***Anaplasma*** **spp.**				
Breed	Exotic	16/62 (25.8)	1.64 (0.84–3.09)	0.132
	Cross	17/74 (23.0)	1.41 (0.74–2.58)	0.281
	Local	50/286 (17.5)	Reference	Overall = 0.251
Sex	Male	42/207 (20.3)	1.08 (0.67–1.75)	0.753
	Female	41/215 (19.1)	Reference	
Age	≥ 12 months	76/369 (20.6)	1.70 (0.79–4.27)	0.187
	< 12 months	7/53 (13.2)	Reference	
BCS	3–5	27/133 (20.3)	1.06 (0.63–1.76)	0.825
	1–2.5	56/289 (19.4)	Reference	
Ticks	Present	56/225 (24.9)	2.09 (1.27–3.50)	0.004**
	Absent	27/197 (13.7)	Reference	
Sampling site	SH	47/203 (23.2)	1.53 (0.95–2.50)	0.083
	LM	36/219 (16.4)	Reference	
***Ehrlichia*** **spp.**				
Breed	Exotic	10/62 (16.1)	2.21 (0.87–5.18)	0.079
	Cross	3/74 (4.1)	0.41 (0.06–1.48)	0.244
	Local	15/286 (5.2)	Reference	Overall = 0.063
Sex	Male	15/207 (7.2)	1.21 (0.56–2.66)	0.621
	Female	13/215 (6.0)	Reference	
Age	≥ 12 months	25/369 (6.8)	1.21 (0.41–5.22)	0.756
	< 12 months	3/53 (5.7)	Reference	
BCS	3–5	7/133 (5.3)	0.71 (0.27–1.64)	0.433
	1–2.5	21/289 (7.3)	Reference	
Ticks	Present	21/225 (9.3)	2.79 (1.22–7.23)	0.015*
	Absent	7/197 (3.6)	Reference	
Sampling site	SH	13/203 (6.4)	0.93 (0.43–2.01)	0.854
	LM	15/219 (6.8)	Reference	
***Theileria*** **spp.**				
Breed	Exotic	2/62 (3.2)	0.20 (0.03–0.67)	0.029*
	Cross	9/74 (12.2)	0.83 (0.36–1.72)	0.630
	Local	41/286 (14.3)	Reference	Overall = 0.023*
Sex	Male	25/207 (12.1)	0.96 (0.53–1.71)	0.881
	Female	27/215 (12.6)	Reference	
Age	≥ 12 months	44/369 (11.9)	0.76 (0.35–1.84)	0.522
	< 12 months	8/53 (15.1)	Reference	
BCS	3–5	21/133 (15.8)	1.56 (0.85–2.82)	0.149
	1–2.5	31/289 (10.7)	Reference	
Ticks	Present	28/225 (12.4)	1.02 (0.57–1.85)	0.935
	Absent	24/197 (12.2)	Reference	
Sampling site	SH	31/203 (15.3)	1.70 (0.95–3.10)	0.076
	LM	21/219 (9.6)	Reference	
***A. marginale***				
Breed	Exotic	11/62 (17.7)	7.50 (2.90–20.24)	< 0.001
	Cross	2/74 (2.7)	0.96 (0.14–3.95)	0.965
	Local	8/286 (2.8)	Reference	Overall < 0.001***
Sex	Male	11/207 (5.3)	1.15 (0.47–2.82)	0.754
	Female	10/215 (4.7)	Reference	
BCS	3–5	5/133 (3.8)	0.67 (0.21–1.74)	0.424
	1–2.5	16/289 (5.5)	Reference	
Ticks	Present	14/225 (6.2)	1.80 (0.73–4.84)	0.203
	Absent	7/197 (3.6)	Reference	
Sampling site	SH	16/203 (7.9)	3.66 (1.40–11.37)	0.007**
	LM	5/219 (2.3)	Reference	

Significant codes: * = < 0.05; ** = < 0.01; *** = < 0.001; LM = livestock market; SH = slaughterhouse

Exotic breeds of cattle were significantly more likely to be infected with *A. marginale* (OR: 7.99, 95% CI: 3.04–22.02, *p* <  0.001), and less likely to be infected with *Theileria* spp. (OR: 0.20, 95% CI: 0.03–0.67, *p* = 0.023), compared to local breeds. Cattle on which ticks were present were significantly more likely to be infected with *Anaplasma* spp. (OR: 2.18, 95% CI: 1.32–3.69, *p* = 0.003) and *Ehrlichia* spp. (OR: 2.79, 95% CI: 1.22–7.23, *p* = 0.022), compared with those that had no ticks (Table 3). Cattle sampled at SHs were more likely to be positive for *Anaplasma* spp. (OR: 1.64, 95% CI: 1.01–2.70, *p* = 0.048) and *A. marginale* (OR: 3.84, 95% CI: 1.43–12.21, *p* = 0.012) compared to those sampled at LMs. There was no significant difference with respect to sampling site on the other investigated TBPs (Table 3).

**Table 3:** Logistic regression analyses results for the occurrence of tick-borne pathogens in cattle and associated predictor variables

Variables	Categories	Odds ratio (95% CI)	***p***-value
***Anaplasma*** **spp.**			
Ticks	Present	2.18 (1.32–3.69)	***0.003***
	Absent	Reference	
Sampling site	SH	1.64 (1.01–2.70)	***0.048***
	LM	Reference	
***Ehrlichia*** **spp.**			
Ticks	Present	2.79 (1.22–7.23)	***0.022***
	Absent	Reference	
***Theileria*** **spp.**			
Breed	Exotic	0.20 (0.03–0.67)	0.029
	Cross	0.83 (0.36–1.72)	0.630
	Local	Reference	*Overall* ***= 0.023***
***A. marginale***			
Breed	Exotic	7.99 (3.04–22.02)	< 0.001
	Cross	1.16 (0.17–4.84)	0.855
	Local	Reference	*Overall* ***= < 0.001***
Sampling site	SH	3.84 (1.43–12.21)	***0.012***
	LM	Reference	

Significant *p*-values are shown in bold italic; *SH* slaughterhouse, *LM* livestock market

## Discussion

In this study we detected the majority of TBPs of economic importance in livestock production in Kenya, including *T. parva*, *B. bigemina* and *A. marginale*. We also detected *E. minasensis* and *A. platys,* whose epidemiology and association with clinical disease in cattle in Kenya is still unclear. Phylogenetically, the detected TBPs were, as would be expected, closely related to strains reported previously in ticks and cattle in Kenya and Uganda, suggesting the possible movement of pathogens across borders within cattle harbouring the tick vectors. The natural resistance of local breeds to TBDs, the importance of tick control, and the potential for LMs and SHs to serve as surveillance points for TBPs are highlighted in our logistic regression analysis where exotic breeds, tick presence and sampling at SHs were associated with the occurrence of TBPs.

The overall prevalence of TBPs in this study was lower than what has been reported in previous studies in Kenya, including studies on two specific dairy farms in Kajiado and Machakos counties [3], calves from western Kenya [7], smallholder livestock systems from western Kenya highlands [5], Machakos County [30, 31], and at a wildlife-livestock interface in Lambwe Valley, western Kenya [33]. A probable explanation for this consistent difference between our study and previous ones is that some of the latter studies used serology, rather than PCR, for determining positivity to TBPs; serology is likely to generate higher prevalence than PCR since it measures historical exposure. Indeed, antibodies to *T. parva* were reported to persist in cattle for about 6 months after initial infection [49]. Higher prevalence of TBPs is also expected in cattle at wildlife-livestock interfaces due to spill over from wildlife, as most of the TBPs have wildlife reservoir hosts such as buffalo [50].

In Uganda, a higher prevalence of TBPs was reported in livestock kept under pastoralism in the Karamoja region [48], while lower *T. parva* infection rates, comparable to those identified in our study, were reported in Tororo District of Eastern Uganda which borders western Kenya [51]. In smallholder livestock systems such as in western Kenya, there is variable immunity to East Coast fever and the mortality ranges from 3 to 20% [4]. However, the disease causes higher mortality rates of 40–80% in pastoral systems [4, 52]. Poor tick control strategies and veterinary seeking behaviour have been attributed to the high incidence of TBPs in pastoralist communities [53]. The prevalence of TBPs identified in this study was also lower than that found in Ethiopia [54] and Cameroon [2], but comparable to that detected in China [55]. In this study, the low prevalence of *B. bigemina* and *A. marginale*, the major causes of bovine babesiosis and anaplasmosis, respectively, in Kenya, was consistent with other studies in the same region [5, 7]. Conversely, higher infection rates have been reported in other ecological zones, which are likely to be more suited to the survival of *Rh. decoloratus*, the major vector of these pathogens in Kenya [3]. Ecology has been shown to influence parasite development in ticks, as higher temperatures are thought to retard or even eliminate the infective stages of *B. bigemina* in *Rhipicephalus* ticks [56]. The climate in our study region is equatorial, hot and humid with maximum temperatures ranging from 27 °C to 32 °C and an annual rainfall ranging between 1350 and 2400 mm [57]. Importantly, these temperatures are considerably higher than those around Nairobi and other highland regions [3] and may therefore hinder parasite development. The higher prevalence of *Anaplasma* spp. and *A. marginale* in cattle sampled at SHs, compared to those from LMs, suggests that cattle owners/traders may seek to dispose of sick animals, presumably from TBP infection, via slaughter rather than trading them at LMs. Concomitantly, butchers at these SHs are likely to benefit from buying sick animals at cheaper prices. However, in this study, the body condition score, which is one of the most important variables considered by buyers and sellers of cattle at these LMs/SHs, was not associated with the occurrence of TBPs.

There was a higher prevalence of the mildly pathogenic non-transforming *Theileria* spp. compared to *T. parva* infections, similar to what has previously been reported in Kenya [7], Uganda [48], and Ethiopia [54]. Non-transforming *Theileria* spp. do not induce proliferation of infected lymphocytes and therefore cause only a mild disease in cattle [58]. These benign species have been shown to play an important role in reducing morbidity and mortality due to the pathogenic *T. parva* in indigenous co-infected cattle [59]. We also report the occurrence of dual infections in some of the positive samples, which is expected as we detected multiple TBP infections in individual ticks collected from the same cattle sampled in this study [26]. Co-infection with both benign and pathogenic species is desirable as the benign *Theileria* spp. are thought to reduce severity of pathogenic species infection via a superinfection mechanism [59]. Correspondingly, these apparently healthy animals at LMs are epidemiologically important as they facilitate the dissemination of TBPs to new areas when they are traded. On the other hand, when both co-infecting species are pathogenic the host’s ability to mount an effective immune response may be impeded, resulting in severe clinical disease [10, 60, 61]. Indeed, the mildly pathogenic *Theileria* spp. have dominated co-infections in previous studies [3, 7, 33, 62].

In this study we detected several *A. platys*-like organisms, which are principally canine pathogens causing cyclic thrombocytopenia in dogs. However, they have recently been found to also infect humans, causing clinical disease [14, 16, 63]. Therefore, SH and LM workers are at risk of infection by this organism through infective tick bites. There has also been widespread detection of *A. platys* in apparently healthy cattle [33, 55, 64]. We speculate that the vector, *Rhipicephalus sanguineus*, may be feeding on both dogs and cattle in regions where they co-exist, such as in our study region where free-roaming dogs have been observed to frequent poorly managed SHs [65]. As a result, this dog-tick-cattle cycle can establish a transmission cycle of *A. platys* that can also potentially involve humans. We also report the occurrence of the recently described *E. minasensis* [66] in cattle blood, which has been shown to cause bovine ehrlichiosis in Brazil [67]. In our study, the positive cattle did not show any apparent clinical disease. In Kenya, *E. minasensis* has also been recently reported in apparently healthy dairy cows, highlighting the need for more studies to determine its clinical relevance in the country [25].

We found that exotic cattle breeds were significantly more likely to be infected with *A. marginale*. Generally, Zebu and indigenous breeds have been reported to be less susceptible to TBPs than exotic breeds, due to their innate resistance and constant exposure to TBP infected tick bites, which regularly primes their immune system [68]. This infection pressure ensures that new-born calves are exposed to the pathogen early before their maternal acquired immunity wanes [30]. Comparatively, the immune system of exotic breeds will be naïve, hence they are more susceptible to the adverse effects of TBPs. Conversely, exotic breeds and cross-bred cattle appeared to be less susceptible to *Theileria* spp. infection compared to local breeds. However, this could be due to the low number of infected exotic cattle, compared to local cattle, included in the regression analysis.

Tick presence was significantly associated with the occurrence of *Anaplasma* spp. and *Ehrlichia* spp., which is expected since ticks are the vectors driving the transmission, and their epidemiology therefore closely mirrors that of TBPs [10, 69]. The stability of endemicity in a given region depends on the suitability of the ecology for the survival of ticks in that area [68]. In our previous study on the same site [26], we collected diverse tick species from the same animals; therefore, this association is most likely to be pronounced for *Anaplasma* spp. and *Ehrlichia* spp. because they are transmitted by several tick species and even mechanically, unlike *T. mutans* and *T. velifera* that are restricted to *Amblyomma* spp. None of the other risk factors that we investigated, such as animal sex and age, were significant predictors of TBP infection.

We did not detect *E. ruminantium*, *Rickettsia* spp., or any viruses such as CCHF. *Ehrlichia ruminantium*, the cause of heartwater in ruminants, is found mostly in endothelial cells causing vasculitis and has very few stages circulating in the blood system during chronic illness. This means that, given its low presence in peripheral blood, it is less likely to be picked up by ticks and other mechanical vectors, hence the low transmissibility [9, 70]. Recent studies have not detected this pathogen in cattle [33], and low detection of *Ehrlichia* spp. in ticks has been reported in previous studies in Kenya [26, 71, 72]. On the other hand, *E. ruminantium*, *B. bigemina* and *T. parva* all cause severe disease, hence cattle infected with these pathogens may not be presented for sale or slaughter in cases of acute disease leading to deaths on the farm. However, our experience at these LMs and SHs has shown us that a variety of cattle are brought for potential trading or slaughter, including some in very poor condition. *Ehrlichia ruminantium* has also been implicated as a potential cause of undifferentiated camel diseases in northern Kenya [73], and has been associated with ticks of tortoises [72] and even with humans [74].

The absence of *Rickettsia* spp., especially *R. africae*, is surprising because a similar study detected a high prevalence of the pathogen in *Am. variegatum* ticks [46], as we previously did in *Am. variegatum* ticks removed from the same animals whose blood samples are analysed here [26]. However, a similar recent study in Kenya also did not report any *Rickettsia* spp. from cattle [33]. Given that *Am. variegatum* ticks are the reservoirs of *R. africae,* these findings may indicate that the ticks may not be efficient vectors of the pathogen and that cattle develop low, transient rickettsaemia and only serve to harbour the tick (*Am. variegatum*) and not the pathogen in the epidemiology of rickettsioses. This is supported by a previous study in Argentina where all of the cattle followed up for 18 months did not have measurable rickettsaemia, but 90% of them were seropositive for *Rickettsia parkeri* a related spotted fever group *Rickettsia*. Additionally, 20% of the ticks removed from these cattle were positive for *R. parkeri* DNA [75]. Therefore, serological surveys may provide more information when studying the exposure of cattle to *R. africae*, compared to molecular methods that target the pathogen’s DNA.

The use of archived samples may have decreased the ability to detect TBPs, especially viruses, in the blood samples after several freeze-thaw cycles. Outside outbreak phases, the viraemia caused by CCHF and Rift Valley fever in reservoir hosts such as cattle may be lower than the PCR detectable limits [76]; hence the combined use of PCR and serology may give a clearer picture of their occurrence. This challenge is also reflected in the low infection rates of these arboviruses in their major vectors, such as ticks and mosquitoes collected from the same sampling sites [26, 77]. Moreover, arboviruses, such as dengue and chikungunya, have limited enzootic cycles and cattle do not seem to be play a key role in their maintenance [78, 79]. To counter the occurrence of false negatives, internal PCR controls that amplify host messenger RNA can be developed and used in future viral analysis. The sample selection also did not permit equal/proportional representation of the sampling sites and other variables in the final batch of selected samples, and subsequently limited the statistical analyses that could be performed. Future studies could employ a matched case-control study design to ensure sufficient numbers of positive cases are included.

While in some cases, samples collected from sentinel sites such as LMs and SHs may not substantially reflect the prevalence of TBPs in the specific localities investigated, our sampling site selection was done purposively to include both small and large sites, which received cattle from the vicinity (within county) and from other neighbouring counties, respectively [80]. Moreover, in a related study in the same region, most of the cattle (99.7%) that were brought to SHs were sourced from within the county [81]. Central point sampling at sites such as at LMs/SHs has also been found to be representative of the actual prevalence of other vector-borne diseases affecting cattle, especially in epidemic foci. Furthermore, central point sampling is more cost-effective and logistically convenient [82].

The use of primers targeting the 16S rRNA region of tick-borne bacteria, even those amplifying long fragments of up to 1000 base pairs, may also have limited our ability to precisely resolve some of the TBPs especially *Anaplasma* spp. Despite these limitations, this study reveals the occurrence (single and dual infections) and diversity of TBPs, and some of the important factors influencing their occurrence, in cattle in western Kenya.

## Conclusions

We detected TBPs of economic and potential zoonotic importance in cattle at LMs and SHs. These findings underline the importance of these sentinel sites in studying the transmission dynamics of cattle TBPs and tick-borne zoonotic pathogens as apparently healthy cattle are traded at these LMs and SHs. The veterinary or zoonotic importance of the recently described *E. minasensis* needs further investigation in the local context, while in future the use of other genetic markers such as the heat shock protein (*groEL*) and major surface protein 4 (*msp4*) genes, in combination with the 16S rRNA marker, may improve the resolution of *A. platys*-like pathogens detected. With increasing reports of the occurrence of this bacterial species in humans elsewhere it is also important to assess *A. platys* presence and its association with clinical cases in humans in Kenya. Furthermore, competence studies on the transmission of *R. africae* by *Am. variegatum* ticks in the local context are required to explain its absence in cattle blood samples. Livestock markets are an important source of subsistence in the livestock production sector, but they can also pose a risk of translocation of apparently healthy but infected cattle to other areas. Given this information, surveillance for TBPs at these sites and the importance of tick control should be emphasized, together with the regulation of cattle movement and trade at these points of livestock concentration.

## Methods

### Study site and livestock sampling

The study was based in western Kenya, on the border with Uganda. The study sites, which included the counties of Busia, Bungoma and Kakamega, lie in the East African Lake Victoria basin, where there is an abundance of livestock raised in smallholder production systems.

This study was part of a larger study to develop an integrated surveillance system for zoonotic diseases in western Kenya using hospitals, LMs and SHs as sentinel sites [80]. Briefly, a sampling framework of all the LMs and SHs in the three counties was established. From this, four LMs and neighbouring SHs in each county were selected for sampling; the selection was based on the animal throughput at each site and their accessibility from Busia town where our field laboratory was located. Each LM and neighbouring SH were visited once every four weeks over a two-year period. During each visit, up to 10 animals were sampled at each site. At LMs, the 10 sampled animals, which included cattle and small ruminants, were randomly selected. At the SHs, all the animals brought to slaughter (if ≤10), or a sub-set of these (if > 10), were sampled, and these included cattle, small ruminants, and pigs.

A clinical examination was performed on each sampled animal, and blood samples were collected from the jugular vein into 10 ml plain, heparinized, and EDTA tubes (BD Vacutainer®) using an 18-gauge rubber-capped needle. The presence of ticks on the animal was also noted. Blood samples were transported to the International Livestock Research Institute (ILRI) field lab in Busia in a cooler box on ice packs, and later shipped on dry ice to the ILRI Nairobi laboratory where they were stored at − 80 °C.

**Fig. 5:**
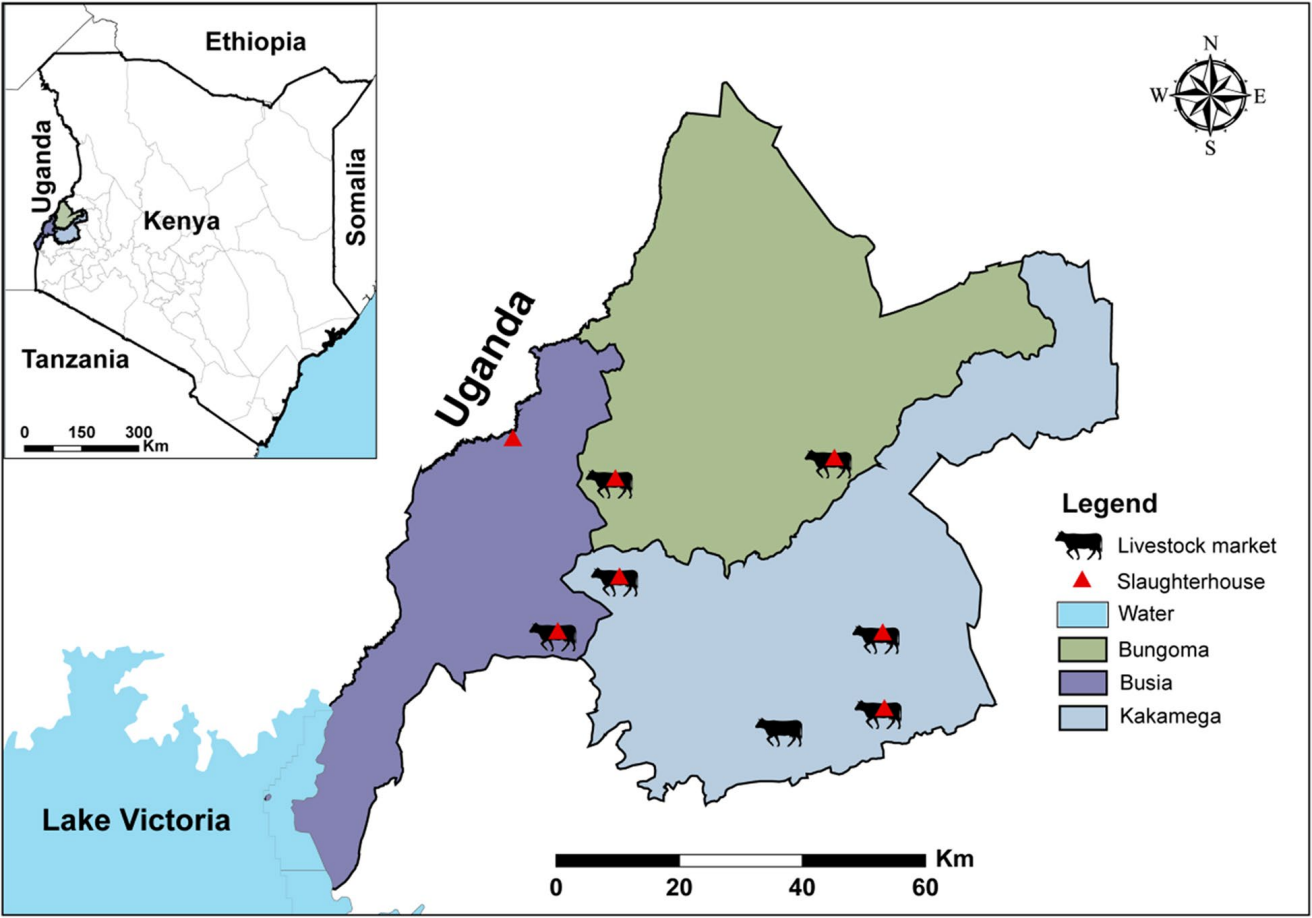
Map of western Kenya showing the three neighbouring counties included in this study. Slaughterhouses and livestock markets from which blood samples used in this study were collected from cattle are shown

In total, 1977 and 1509 animals were sampled at the LMs and SHs, respectively; of these, 1293 and 885 were cattle [80]. For this study, we included blood samples and associated meta-data of 422 cattle which were sampled in seven LMs (*n* = 219) and seven SHs (*n* = 203) in the three counties (Fig. 5). Selection criteria for these 422 samples analysed in this study included: (i) cattle sampled between May 2017 and January 2019, for which TBPs (viral/bacteria/protozoa) data were available [26], (ii) availability of complete meta-data, and (iii) blood volumes adequate for the envisaged analyses. Furthermore, because CCHF virus positive ticks were detected in Kakamega [26], samples from Kakamega County were prioritized. Selected samples were thawed, aliquoted and transported on ice to the Martin Lüscher Emerging Infectious Disease (ML-EID) laboratory at the International Centre of Insect Physiology and Ecology (*icipe*) where subsequent analyses were performed.

### Nucleic acid extraction

We extracted both DNA and RNA from blood using the TRIzol™ reagent standard protocol (Thermofisher, USA). Dengue serotype 2 and Sindbis virus cultured on Vero cell lines in a previous study were included in each RNA extraction run [83]. After nucleic acid extraction, 5 μl of the total RNA was subjected to cDNA synthesis using a High Capacity cDNA Reverse Transcription (RT) kit (Life Technologies, USA). The ‘10 μl’ reaction mixtures contained 1X RT buffer, 4 mM dNTPs, 600 μM random hexamers [84], 2.5 U/μl reverse transcriptase enzyme, and 1 U/μl RNAse inhibitor.

### Molecular detection of arboviral, bacterial, and protozoan pathogens

#### Detection of arboviruses

An established multiplex PCR coupled with high-resolution melting (HRM) analysis was used to screen blood samples for arboviruses [83]. The reaction mixture contained 1 μl of cDNA template, 5 μl of 2x MyTaq HS Mix (Bioline, UK), 1 μl of 50 μM SYTO-9 (Life Technologies, USA) and degenerate primer mix (Table 4). The amplicons were subjected to HRM following amplification with previously described thermal cycling conditions [83]. End-reaction melting profiles were visually inspected to identify samples with melt peaks representing specific amplification.

**Table 4:** Primers that were used for the detection of arboviruses, tick-borne bacteria and protozoa

Target gene	Primer name	Primer sequence (5′ – 3′)	Product size (bp)	References
*Phlebovirus* NP (S-segment)	Phlebo JV3a F	AGTTTGCTTATCAAGGGTTTGATGC	150	[83]
	Phlebo JV3b F	GAGTTTGCTTATCAAGGGTTTGACC		
	Phlebo JV3 R	CCGGCAAAGCTGGGGTGCAT		
*Nairovirus* RdRp (L-segment)	Nairo L 1a F	TCTCAAAGATATCAATCCCCCCITTACCC	150	[83]
	Nairo L 1b F	TCTCAAAGACATCAATCCCCCTTWTCCC		
	Nairo L 1a R	CTATRCTGTGRTAGAAGCAGTTCCCATC		
	Nairo L 1b R	GCAATACTATGATAAAAACAATTMCCATCAC		
	Nairo L 1c R	CAATGCTGTGRTARAARCAGTTGCCATC		
	Nairo L 1d R	GCAATGCTATGGTAGAAACAGTTTCCATC		
*Alphavirus* NS4	Vir 2052 F	TGGCGCTATGATGAAATCTGGAATGTT	150	[85]
	Vir 2052 R	TACGATGTTGTCGTCGCCGATGAA		
*Flavivirus* NS5	Flavi JV2a F	AGYMGHGCCATHTGGTWCATGTGG	150	[83]
	Flavi JV2b F	AGCCGYGCCATHTGGTATATGTGG		
	Flavi JV2c F	AGYCGMGCAATHTGGTACATGTGG		
	Flavi JV2d F	AGTAGAGCTATATGGTACATGTGG		
	Flavi JV2a R	GTRTCCCADCCDGCDGTRTCATC		
	Flavi JV2b R	GTRTCCCAKCCWGCTGTGTCGTC		
*Orthobunyavirus* NP (s-segment)	Bunyagroup F	CTGCTAACACCAGCAGTACTTTTGAC	210	[86]
	Bunyagroup R	TGGAGGGTAAGACCATCGTCAGGAACTG		
Dhori virus NP	Dhori F	CGAGGAAGAGCAAAGGAAAG	200	[83]
	Dhori R	GTGCGCCCCTCTGGGGTTT		
Thogoto virus (M-segment)	Thogoto S6 F	GATGACAGYCCTTCTGCAGTGGTGT	200	[83]
	Thogoto S6 R	RACTTTRTTGCTGACGTTCTTGAGGAC		
*Rickettsia* 16S rRNA	Rick-F	GAACGCTATCGGTATGCTTAACACA	364	[87]
	Rick-R	CATCACTCACTCGGTATTGCTGGA		
*Theileria* and *Babesia* 18S rRNA	RLB-F	GAGGTAGTGACAAGAAATAACAATA	450	[88]
	RLB-R	TCTTCGATCCCCTAACTTTC		
*Anaplasma* 16S rRNA	*Anaplasma*JV F	CGGTGGAGCATGTGGTTTAATTC	300	[71]
	*Anaplasma*JV R	CGRCGTTGCAACCTATTGTAGTC		
*Ehrlichia* 16S rRNA	*Ehrlichia* 16S F	CGTAAAGGGCACGTAGGTGGACTA	200	[89]
	*Ehrlichia* 16S R	CACCTCAGTGTCAGTATCGAACCA		
*Anaplasma/Ehrlichia* 16S rRNA	EHR16SD	GGTACCYACAGAAGAAGTCC	1090	[90–92]
	pH 1522	AAGGAGGTGATCCAGCCGCA		
	pH 1492	GGCTACCTTGTTACGACTT		

### Detection of bacterial and protozoan pathogens

Using a combination of PCR-HRM and conventional PCR, previously developed genus-specific methods and primers were used to detect *Rickettsia*, *Anaplasma*, and *Ehrlichia* 16S rRNA, and *Theileria* and *Babesia* 18S rRNA gene sequences. Ten-microliter reactions that consisted of 2 μl template, 2 μl 5X HOT FIREPol® EvaGreen HRM Mix (Solis BioDyne, Estonia) and 0.5 μM of each primer were constituted for the PCR-HRM. Positive controls of *Anaplasma*, *Rickettsia*, *Theileria*, and *Babesia* spp. previously detected in clinical samples banked in *icipe*’s ML-EID lab were included in the runs. Second stage amplification of positive samples utilised different primers to generate larger PCR products where possible (Table 4) for sequencing and phylogenetic inference purposes.

Positive *Ehrlichia* spp. and *Anaplasma* spp. samples were further amplified with a conventional semi-nested PCR using the following touchdown PCR cycling conditions: for primary amplification, a hot-start activation step of 95 °C of 15 min was followed by 1 cycle at an annealing temperature (Ta) of 63 °C for 30 s, 2 cycles at a Ta of 62 °C for 30 s, 2 cycles at a Ta of 61 °C for 30 s, and 35 cycles at a Ta of 60 °C for 30 s. Each of these annealing steps was preceded by a denaturation step of 95 °C for 20 s, and followed by primer extension at 72 °C for 80 s, with the final extension being conducted at 72 °C for 10 min. The secondary amplification utilized 2 μl PCR products from the primary reactions in 20 μl reactions. The cycling profile consisted of 95 °C for 15 min; 3 cycles of 95 °C for 20 s, 61 °C for 30 s, and 72 °C for 90 s; 37 cycles of 95 °C for 20 s, 60 °C for 30 s, and 72 °C for 80 s, and a final extension at 72 °C for 10 min. All amplicons were visualized by 1.5% agarose gel electrophoresis and representative amplicons were purified (Exo 1-rSAP, Biolabs, UK) and sequenced in both directions at Macrogen (The Netherlands).

### Phylogenetic analysis

Sequences were inspected and edited in Geneious prime version 2019.0.4 software (created by Biomatters, Auckland, New Zealand). Sequence contigs were then queried against known sequences in the GenBank nr database (http://www.ncbi. nlm.nih.gov/) using BLAST to confirm their identity and relation to existing deposited sequences [93]. Study sequences were then aligned with related pathogen sequences available in the GenBank nr database using the MAFFT plugin in Geneious Prime software version 2020.2.2 [94]. Maximum-likelihood phylogenies were constructed using PhyML v. 3.0 with automatic model selection based on Akaike information criterion. Tree topologies were estimated over 1000 bootstrap replicates with nearest neighbour interchange improvements [95]. Trees were visualized and edited in FigTree 1.4.4 [96].

### Statistical analysis

Logistic regression in R® version 4.0.3 was performed using cattle breed, sex, age, body condition score, presence/absence of ticks, and sampling site as predictor variables, and the PCR-based positivity of cattle to *Anaplasma* spp., *Ehrlichia* spp., *Theileria* spp., and *A. marginale* as the response variables. Based on the metadata accompanying each sample, cattle breed was recoded into three levels for the analysis; animals recorded as zebu, indigenous, shorthorn, or shorthorn x zebu were classified as local, animals recorded as local x grade were classified as cross, and animals recorded as grade or exotic were classified as exotic. Sex (male/female) was used as recorded, while age, recorded as a continuous variable in months, was dichotomized into cattle less than 12 months old, and cattle aged 12 months and above. Body condition score, which was recorded on a scale of 1 (thin) to 5 (obese), was dichotomized into 1–2.5 (lower range) and 3–5 (upper range). Tick presence or absence, which was determined by visual inspection of predilection sites, was kept as recorded (Yes/No) for the analysis. Sampling site (LM/SH) was used as recorded.

The association of these variables with TBP occurrence was determined by estimating odds ratios, confidence intervals, and *P*-values. We first performed univariable analysis between the predictor variables and each of the TBP concerned, and then selected all the variables with a univariable likelihood ratio test *P*-value < 0.1 for inclusion into the multivariable models. In the multivariable model a Wald *P*-value of < 0.05 was considered statistically significant.

## Data Availability

The dataset generated and analysed in this study can be made available from the corresponding authors on reasonable request. All the nucleotide sequences generated from this study have been deposited and are available in the GenBank database under the accession numbers indicated in text.

## Abbreviations

TBP: Tick-borne pathogen;
TBD: Tick-borne disease
CCHF: Crimean-Congo haemorrhagic fever
LM: Livestock market
SH: Slaughterhouse
PCR-HRM: Polymerase chain reaction-high resolution melting
ICIPE: International Centre of Insect Physiology and Ecology
ML-EID: Martin Lüscher Emerging Infectious Diseases
ILRI: International Livestock Research Institute
IREC: Institutional Animal Care and Use Committee
CI: Confidence interval
OR: Odds ratio
DNA: Deoxyribonucleic acid
RNA: Ribonucleic acid
cDNA: Complementary deoxyribonucleic acid
BLAST: Basic Local Alignment Search Tool
MAFFT: Multiple alignment using fast Fourier transform
dNTPs: deoxyribonucleotide triphosphate
16S rRNA: 16S ribosomal ribonucleic acid
18S rRNA: 18S ribosomal ribonucleic acid
NACOSTI: National Commission for Science, Technology and Innovation
